# Role of peroxisome proliferators-activated receptor-gamma in advanced glycation end product-mediated functional loss of voltage-gated potassium channel in rat coronary arteries

**DOI:** 10.1186/s12872-020-01613-y

**Published:** 2020-07-14

**Authors:** Side Gao, Bing Hua, Qingbo Liu, Huirong Liu, Weiping Li, Hongwei Li

**Affiliations:** 1 Department of Cardiology, Cardiovascular Center, Beijing Friendship Hospital, Capital Medical University, 95 Yong An Road, Xicheng, Beijing, 100050 P. R. China; 2 School of Basic Medical Sciences, Capital Medical University, Beijing, 100069 P. R. China; 3 Beijing Key Laboratory of Metabolic Disorder Related Cardiovascular Disease, Beijing, 100069 P. R. China; 4 Department of Internal Medicine, Medical Health Center, Beijing Friendship Hospital, Capital Medical University, Beijing, 100050 P. R. China

**Keywords:** Advanced glycation end product, Voltage-gated K^+^ channel, Coronary dilation, Smooth muscle cells, PPAR-γ pathway

## Abstract

**Background:**

High blood glucose impairs voltage-gated K^+^ (Kv) channel-mediated vasodilation in rat coronary artery smooth muscle cells (CSMCs) via oxidative stress. Advanced glycation end product (AGE) and receptor for AGE (RAGE) axis has been found to impair coronary dilation by reducing Kv channel activity in diabetic rat small coronary arteries (RSCAs). However, its underlying mechanism remain unclear. Here, we used isolated arteries and primary CSMCs to investigate the effect of AGE incubation on Kv channel-mediated coronary dilation and the possible involvement of peroxisome proliferators-activated receptor (PPAR) -γ pathway.

**Methods:**

The RSCAs and primary CSMCs were isolated, cultured, and treated with bovine serum albumin (BSA), AGE-BSA, alagrebrium (ALA, AGE cross-linking breaker), pioglitazone (PIO, PPAR-γ activator) and/or GW9662 (PPAR-γ inhibitor). The groups were accordingly divided as control, BSA, AGE, AGE + ALA, AGE + PIO, or AGE + PIO + GW9662. Kv channel-mediated dilation was analyzed using wire myograph. Histology and immunohistochemistry of RSCAs were performed. Western blot was used to detect the protein expression of RAGE, major Kv channel subunits expressed in CSMCs (Kv1.2 and Kv1.5), PPAR-γ, and nicotinamide adenine dinucleotide phosphate (NADPH) oxidase-2 (NOX-2).

**Results:**

AGE markedly reduced Forskolin-induced Kv channel-mediated dilation of RSCAs by engaging with RAGE, and ALA or PIO significantly reversed the functional loss of Kv channel. In both RSCAs and CSMCs, AGE reduced Kv1.2/1.5 expression, increased RAGE and NOX-2 expression, and inhibited PPAR-γ expression, while ALA or PIO treatment partially reversed the inhibiting effects of AGE on Kv1.2/1.5 expression, accompanied by the downregulation of RAGE and decreased oxidative stress. Meanwhile, silencing of RAGE with siRNA remarkably alleviated the AGE-induced downregulation of Kv1.2/1.5 expression in CSMCs.

**Conclusion:**

AGE reduces the Kv channel expression in CSMCs and further impairs the Kv channel-mediated dilation in RSCAs. The AGE/RAGE axis may enhance oxidative stress by inhibiting the downstream PPAR-γ pathway, thus playing a critical role in the dysfunction of Kv channels.

## Background

Advanced glycation end product (AGE), a group of derivatives yielded by nonenzymatic glycation of proteins, has been recognized as a key pathogenic factor in diabetic vasculopathy and thereby an important predictor for poor cardiovascular outcomes [1]. Previous studies have shown that AGE exerts detrimental effects through direct cross-linking of target proteins and indirect ways by activating downstream signaling pathways. In the latter, AGE, along with its receptor (RAGE), can markedly induce inflammation and enhance oxidative stress in vascular smooth muscle cells (VSMCs) and endothelial cells, and may further lead to a broad spectrum of vascular damages, including atherosclerotic plaque formation, calcification, arterial stiffness, and/or vasodilator dysfunction [2]. This insight is particularly relevant in diabetic populations who exhibit early-onset myocardial ischemic symptoms and increased risks of death [3].

Among the well-known regulators of vasomotor function, voltage-gated K^+^ (Kv) channels play a pivotal role in maintaining the resting membrane potential of VSMCs and vascular tone under physiological and pathophysiological conditions [4–7]. It’s reported that hyperglycemia or excessive formation of AGE impairs the Kv1.2 and Kv1.5 channels in coronary SMCs (CSMCs) through oxidative stress [8–10] and further damages the Kv channel-mediated coronary dilation in diabetic rats [11–13]. Meanwhile, treatment with AGE cross-linking breaker alagrebrium (ALA) largely ameliorates the vasodilator dysfunction of mesenteric arteries in diabetic rats [14, 15]. A recent study proved that hydrogen peroxide-induced dilation of human adipose arterioles was markedly reduced in patients with coronary artery disease, accompanied by a loss of Kv1.5-dependent dilation, indicating that the function of Kv channel is redox regulated in VSMCs [16].

Peroxisome proliferators-activated receptor-gamma (PPAR-γ), a key transcription factor that regulates adipogenesis, lipid metabolism, and insulin sensitization, has been proved to be linked with AGE/RAGE axis and to exert its protective effect on vascular beds in the setting of diabetes by inhibiting vascular inflammation and oxidative stress [17]. Recent evidence suggests that PPAR-γ activator pioglitazone could reduce high glucose-induced RAGE expression and inhibit the nicotinamide adenine dinucleotide phosphate (NADPH) oxidase-mediated reactive oxygen species (ROS) generation in CSMCs [18].

So far, many studies have confirmed the detrimental effects of AGE on the endothelium-mediated vasodilation [19, 20], however, its effect on the Kv channel in CSMCs has not been fully elucidated. Here, we tested the hypothesis that AGE would impair Kv channel-mediated SMC-dependent dilation of rat small coronary arteries (RSCAs), and further explored the potential role of PPAR-γ pathway and oxidative stress in the AGE-induced functional loss of Kv channel.

## Methods

### Animals

Male Sprague-Dawley rats (8-week-old, 180-200 g weight) were provided by Vital River Laboratory Animal Technology (Beijing, China). They were housed under specific pathogen-free conditions and given free access to food and water. All animal experiments were performed based on the National Institutes of Health guidelines for care and use of laboratory animals, and were approved by the Animal Care and Use Committee of Capital Medical University.

### Preparation and treatment of isolated RSCAs

Rats were anesthetized with sodium pentobarbital (60 mg/kg i.p.) and were sacrificed when the heart was removed. RSCAs (internal diameter 150-200 μm) were carefully dissected from surrounding myocardium and immediately placed in ice-cold Hanks solution. The arteries were cleared of fat and connective tissue and were cut into 2-mm long rings. The endothelium was denuded by dry air, and the denudation was verified by failure to dilate in response to 1 μM (mol/L) acetylcholine. For isometric force measurement, arteries were directly mounted in the myograph chamber containing HEPES-buffered solution. For protein level’s detection, arteries were incubated in Dulbecco’s modified Eagle’s medium (DMEM) containing 1 g/L glucose, 10% fetal bovine serum (FBS), 100 U/mL penicillin, and 100 mg/mL streptomycin at 37 °C for 24 h.

The effects of ALA (AGE cross-linking breaker), PIO (PPAR-γ activator), and GW9662 (PPAR-γ inhibitor) on the AGE-induced impairment of Kv channel in RSCAs were evaluated. The arteries were divided into the following treatment groups: vehicle (DMEM group), 200 μg/mL BSA (BSA group), 200 μg/mL AGE-BSA (AGE group), 200 μg/mL AGE-BSA + 1 mM ALA (AGE + ALA group), 200 μg/mL AGE-BSA + 0.1 mM PIO (AGE + PIO group), and 200 μg/mL AGE-BSA + 0.1 mM PIO + 0.1 mM GW9662 (AGE + PIO + GW9662 group). The concentrations of AGE and other reagents were based on previous published studies [14, 15, 21].

### Isometric force measurements and Myograph protocol

Freshly isolated arterial rings of RSCAs were threaded onto two stainless steel wires (40 μm in diameter) and were mounted in the 5 mL chambers of a multi-myograph system (model 610 M, DMT, Aarhus, Denmark) containing HEPES-buffered solution (in g/L: 8.415 NaCl, 0.432 KCl, 0.244 MgCl_2_, 0.277 CaCl_2_, 2 glucose, 1.1915 HEPES) continuously gassed with a mixture of 95% O_2_ and 5% CO_2_ at 37 °C for isometric force measurement (Supplementary Fig. 1). Tension signals were recorded by the PowerLab and saved to LabChart (ADInstruments, Ltd., Aarhus, Denmark). The standardization procedure was performed as previously described by McPherson [22], and the transmural pressure was set to a baseline value of 100 mmHg. Then, the arteries were equilibrated for 2 h in HEPES-buffered solution containing vehicle, BSA (200 μg/mL), AGE-BSA (200 μg/mL), AGE-BSA + ALA (1 mM), AGE-BSA + PIO (0.1 mM), or AGE-BSA + PIO + GW9662 (0.1 mM). After incubation, vessel viability was checked twice by administration of 60 mM KCl. When tension comes to baseline after washout, the arteries were precontracted with 1 μM U46619 (a vasoconstrictor analog of thromboxaneA_2_), and Forskolin (cyclic adenosine monophosphate activator) with a concentration from 10^− 10^ M to 10^− 5^ M was used to induce an endothelium-independent vasodilation (Supplementary Fig. 2). After further washout and equilibration, the vessels were treated with 3 mM 4-aminopyridine (4-AP, a broad-spectrum Kv channel blocker) for 30 min and the Forskolin-induced vasodilation was repeated after precontraction with 1 μM U46619. In this study, Kv channel-mediated relaxation refers to the relaxation which can be blocked by 4-AP, that is the difference between maximum dilation measured before and after incubation with 4-AP.

### Western blot

Total protein was extracted from RSCAs or the supernatant of cell lysate of CSMCs after incubation in vitro. Protein concentration was determined using a BCA protein assay kit. Equal amounts of protein were separated by 10% SDS-PAGE, transferred to PVDF membranes, and stained with the following primary antibodies (all from Abcam, Cambridge, UK): polyclonal rabbit anti-RAGE (1:500), monoclonal mouse anti-Kv1.2 (1:1000), monoclonal mouse anti-Kv1.5 (1:1000), monoclonal rabbit anti-PPAR-γ (1:1000), monoclonal rabbit anti-NOX-2 (1:1000), and anti-β-actin (1:10000), followed by incubation with HRP-conjugated goat anti-mouse IgG antibody (1:20000, Proteintech Group Inc., IL, USA). Signals were visualized and quantified by a Western Blotting Imaging System (Clinx Science Instruments Co., Shanghai, China). Original Western blot images of target proteins are shown in another supplementary material (Supplementary Figs. 1 to 2 for original images in RSCAs, Supplementary Figs. 3 to 5 in CSMCs, Supplementary Figs. 6 to 7 in cells with RAGE siRNA transfection).

### Histology and immunohistochemistry

The incubated RSCAs were fixed with 4% paraformaldehyde and cross-sectioned. HE and Masson staining were performed. Standard immunohistochemistry protocols were applied. The sections were probed with primary antibody against RAGE (1:200; Abcam) and the biotinylated goat anti-rabbit IgG was used as the secondary antibody. Sections were visualized with diaminobenzidine and counterstained with HE. Images were captured digitally and analyzed using the IMS imaging processing system (Jierdun Biotech, Shanghai, China). Positively stained regions were identified and analyzed. Cardiomyocytes were excluded.

### Cell culture

The RSCAs were obtained with endothelium carefully denuded and adventitia dissected. Primary CSMCs were isolated enzymatically as previous described [8]. Cells were cultured in DMEM for 24 h at 37 °C. The culture medium was changed when the cells were fully adherent, and the cell passages were performed when cell confluency reached 80%. The morphology and growth characteristics of the cells were typical of SMCs and were identified as SMCs by α-smooth muscle actin staining using immunofluorescence (Supplementary Fig. 3). The cells were then used for drug exposure experiments. Similar with the arteries, CSMCs were treated with vehicle (DMEM group), 100 μg/mL BSA (BSA group), 100 μg/mL AGE-BSA (AGE group), 100 μg/mL AGE-BSA + 10 μM ALA (AGE + ALA group), 100 μg/mL AGE-BSA + 1 μM PIO (AGE + PIO group), and 100 μg/mL AGE-BSA + 1 μM PIO + 1 μM GW9662 (AGE + PIO + GW9662 group) at 37 °C for 24 h. The concentrations of the above agents were adopted according to previous published studies [13, 18, 23].

### siRNA transfection

Silencer Select Pre-Designed Small interfering RNAs (siRNAs) against the *AGER* gene of *Rattus norvegicus* were ordered from Invitrogen (CA, USA). The CSMCs were transfected with the siRNA in vitro using lipid-mediated Lipofectamine 3000 (Invitrogen) according to the manufacturer’s protocols. The CSMCs were plated in 6-well plates at 24 h before transfection and were transfected with 75 pmol of siRNA per 6-well plate and used at 48 h after transfection. A scrambled siRNA (Invitrogen) was used as a non-targeting negative control.

### Chemicals

AGE-BSA was purchased from Abcam (Cambridge, UK). Endotoxin levels were found to be less than 0.8 EU/mg protein with the Limulus amebocyte assay (E-Toxate kit, Sigma, MO, USA). ALA was obtained from Shanghai Biopharmaleader (Shanghai, China). All the other chemicals were purchased from Sigma (MO, USA).

### Statistics analysis

Data were presented as means ± standard deviation. Statistical analysis was performed with SPSS 20.0 software (IMB, NY, USA). Comparisons among groups were performed using one-way analysis of variance or the Student’s t-test. The Bonferroni correction was applied when comparing three or more groups. The statistically significance was regarded as *P* < 0.05.

## Results

### AGE impairs Kv channel-mediated vasodilation in RSCAs

**Fig. 1:**
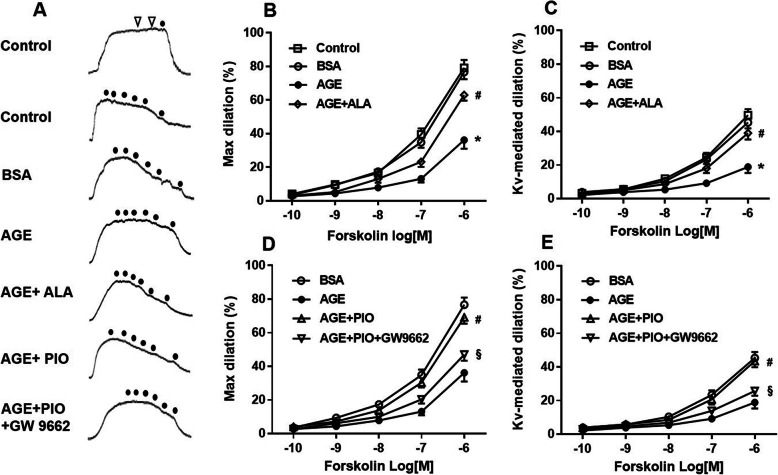
Role of PPAR-γ in AGE-induced impairment of Kv channel-mediated dilation in RSCAs. **a** The first top record of tension indicated that 1 μM or 10 μM acetylcholine (arrows) failed to dilate denuded arteries while 10 μM Forskolin (dot) normally dilated the arteries. The other typical records showed concentration-dependent dilation to Forskolin in endothelium-denuded coronary arteries incubated with control vehicle (HEPES solution), 200 μg/mL BSA, 200 μg/mL AGE, 200 μg/mL AGE+ 1 mM ALA, 200 μg/mL AGE+ 0.1 mM PIO, or 200 μg/mL AGE+ 0.1 mM PIO + 0.1 mM GW9662 for 2 h. Arteries were precontracted with 1 μM U46619. Dots indicate Forskolin concentration from 10^− 10^ to 10^− 5^ M. **b-e** Cumulative concentration-response curves showing Forskolin-induced general dilation (**b**, **d**) and Kv channel-mediated dilation (**c**, **e**) in endothelium-denuded RSCAs. The Kv channel-mediated dilation was defined as the difference between dilations measured before and after incubation with 3 mM 4-aminopyridine (4-AP) for 30 min. *n* = 6 vessels per group. * *P* < 0.05 vs. BSA; # *P* < 0.05 vs. AGE; § *P* < 0.05 vs. AGE + PIO

Isometric force of RSCAs in response to vasoactive agents was measured. Representative records (Fig. 1a**)** illustrate that 1 μM U46619 induced a constant maximal contraction, while Forskolin from 10^− 10^ to 10^− 5^ M elicited a concentration-dependent vasodilation. A high dose of acetylcholine failed to dilate the arteries after endothelial denudation. The dilation was largely depressed by 4-AP, indicating the critical role of Kv channel in the Forskolin-induced dilation.

We investigated the effect of AGE on Kv channel-mediated dilation in RSCAs. As shown in Fig. 1b, similar dilation was observed in control and BSA groups, suggesting that BSA did not affect the vasodilator response to Forskolin. By contrast, coronary relaxation was markedly reduced in the arteries exposed to AGE. The differences in maximal Forskolin-induced dilation between BSA and AGE groups were significant when the concentration of Forskolin was 10^− 7^ M and 10^− 6^ M (Supplementary Table 1). The impaired relaxation, however, could be reversed by ALA. After treatment with 4-AP, new dose-response curves were repeated to ascertain the contribution of Kv channel to Forskolin-elicited dilation (Fig. 1c). There were no significant differences between control and BSA groups. Of note, AGE remarkably attenuated the Kv channel-mediated dilation, while ALA largely restored the AGE-induced functional loss of Kv channel. These findings demonstrate that the impaired relaxation of RSCAs induced by AGE was associated with the vasodilator dysfunction of Kv channel.

### PPAR-γ is involved in AGE-induced impairment of Kv channel-mediated vasodilation

Given excessive formation of AGE can directly enhance oxidative stress in arterial wall, we further explored the role of a redox-sensitive transcription factor, PPAR-γ, in the Kv channel dysfunction induced by AGE. PIO with or without GW9662 were used to treat arteries in the presence of AGE. As shown in Fig. 1d, the Forskolin-induced dilation was largely restored in arteries when co-treated with AGE and PIO as compared with the AGE group. However, this ameliorative effect brought by PIO was offset by additional treatment with GW9662. The effect of PPAR-γ modulation on Kv channel dysfunction was also confirmed (Fig. 1e). We found that PIO significantly reversed the AGE-induced impairment of Kv channel-mediated dilation whereas GW9662 attenuated this reversal. These data indicate that inhibition of PPAR-γ may contribute to the functional loss of Kv channel in RSCAs exposed to AGE.

### Effect of AGE on Kv channel impairment in RSCAs

The Kv1.2 and K1.5 channel are two major subunits of “Shaker-type” Kv1 channel family. They are abundantly expressed in coronary smooth muscles and their activities are susceptible to oxidative stress level in arteries. Therefore, we detected the protein expression of Kv1.2/1.5, RAGE, PPAR-γ, and the critical NADPH oxidase subunit (NOX-2) in both RSCAs and CSMCs.

**Fig. 2:**
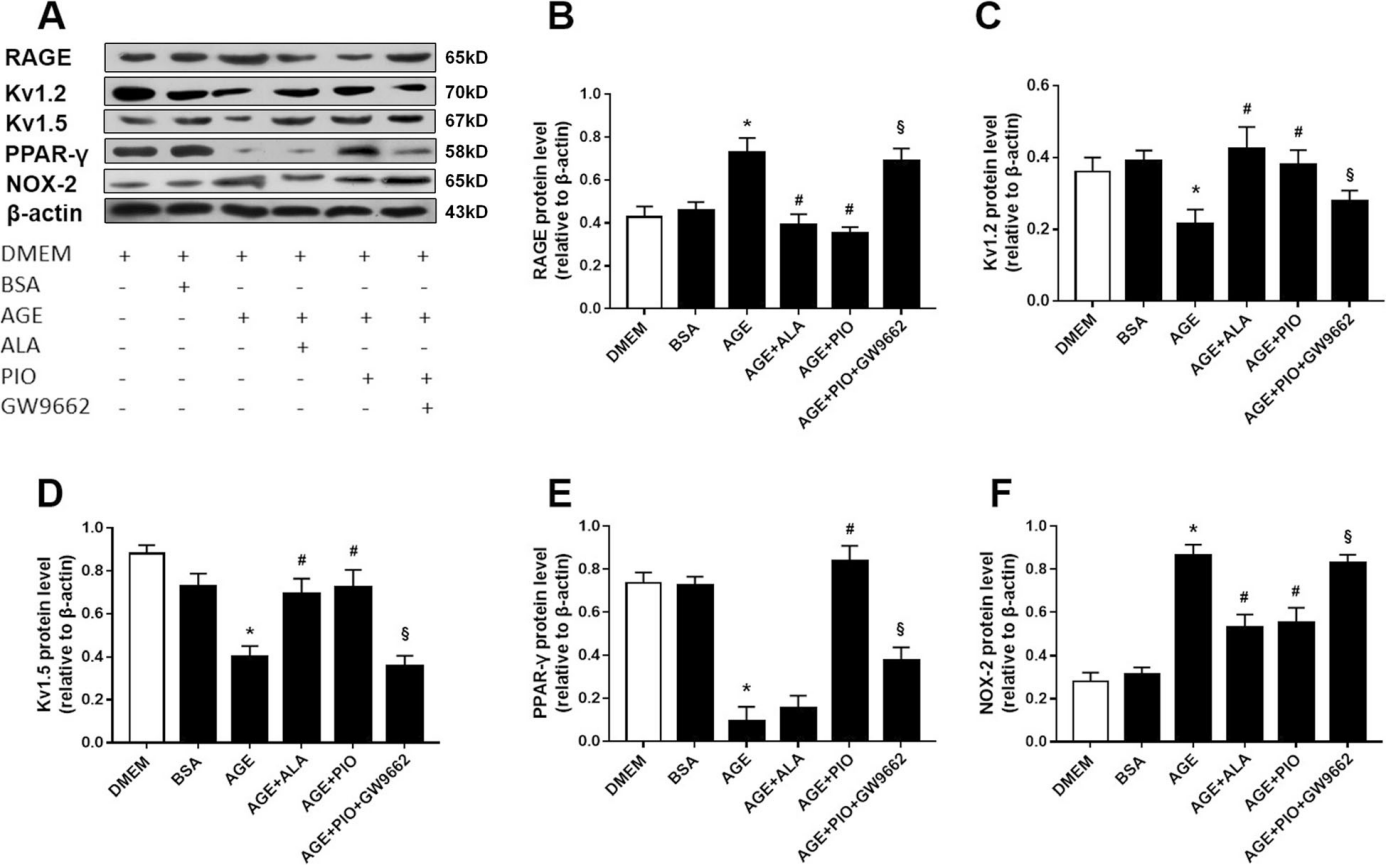
Protein expressions in incubated RSCAs detected by Western blot. The RSCAs were incubated with DMEM, 200 μg/mL BSA, 200 μg/mL AGE, 200 μg/mL AGE+ 1 mM ALA, 200 μg/mL AGE+ 0.1 mM PIO, or 200 μg/mL AGE + 0.1 mM PIO + 0.1 mM GW9662 for 24 h. **a** Representative blots for RAGE, Kv1.2, Kv1.5, PPAR-γ and NOX-2. β-actin was used as an internal reference. **b-d** Mean data for protein expression levels. *n* = 3 per group. * *P* < 0.05 vs. BSA; # *P* < 0.05 vs. AGE; § *P* < 0.05 vs. AGE + PIO

**Fig. 3:**
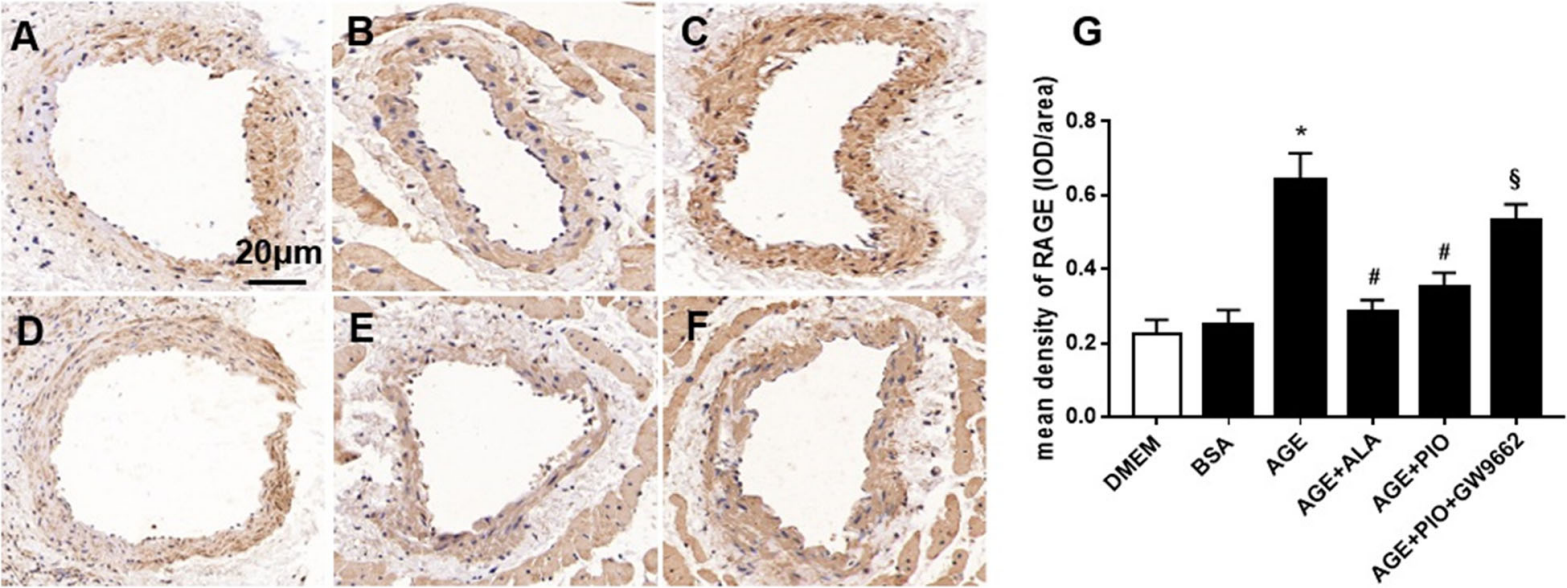
Immunohistochemistry showing protein expression of RAGE in RSCAs. **a-f** Representative immunohistochemical images showing cross-sectioned RSCAs stained with RAGE after incubation. Brown staining is indicative of the expression of RAGE. **g** Mean protein expression of RAGE was shown as IOD/area. **a** DMEM; **b** BSA; **c** AGE; **d** AGE + ALA; **e** AGE + PIO; **f** AGE + PIO + GW9662. *n* = 3 per group. * *P* < 0.05 vs. BSA; # *P* < 0.05 vs. AGE; § *P* < 0.05 vs. AGE + PIO. Magnification: × 200

Western blot analyses (Fig. 2) of RSCAs showed that Kv1.2/1.5 expression was similar in control and BSA group, while AGE significantly reduced Kv1.2/1.5 protein levels as compared with BSA. Meanwhile, AGE markedly increased RAGE and NOX-2 expression and reduced PPAR-γ expression. The above effects induced by AGE could be largely reversed by additional treatment with ALA or PIO (Fig. 2). No obvious arterial structural abnormalities or fibrosis in media layer were observed in sections of RSCAs (Supplementary Figs. 4 & 5). The immunohistochemical staining (Fig. 3) revealed that AGE increased the expression of RAGE, and this AGE-mediated RAGE overexpression was reversed by ALA or PIO.

### Effect of AGE on Kv channel impairment in CSMCs

**Fig. 4:**
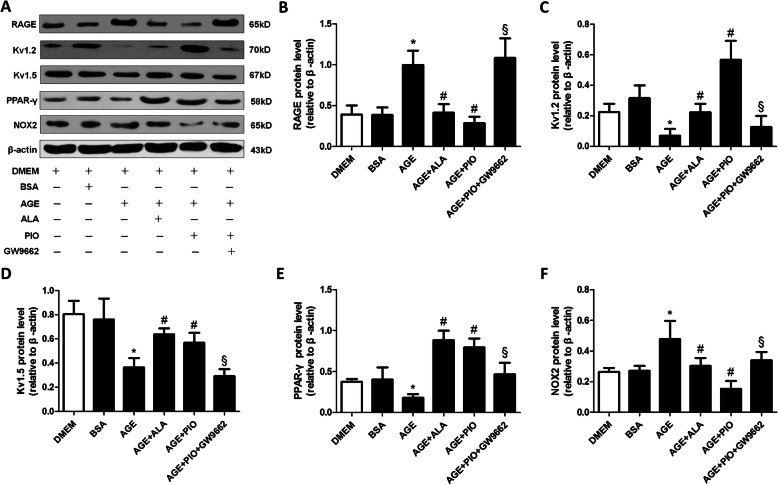
Protein expressions in cultured CSMCs detected by Western blot. The CSMCs were incubated with DMEM, 100 μg/mL BSA, 100 μg/mL AGE, 100 μg/mL AGE+ 10 μM ALA, 100 μg/mL AGE+ 1 μM PIO, or 100 μg/mL AGE+ 1 μM PIO + 1 μM GW9662 for 24 h. **a** Representative blots for RAGE, Kv1.2, Kv1.5, PPAR-γ and NOX-2. β-actin was used as an internal reference. **b-d** Mean data for protein expression levels. *n* = 3 per group. * *P* < 0.05 vs. BSA; # *P* < 0.05 vs. AGE; § *P* < 0.05 vs. AGE + PIO

**Fig. 5:**
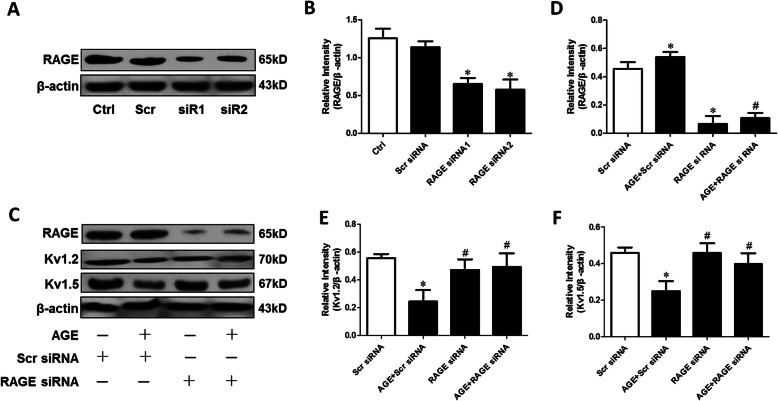
Effect of RAGE silencing on Kv1.2/1.5 expression in CSMCs exposed to AGE. The CSMCs were transfected with RAGE siRNA and the effects of AGE on Kv1.2 and Kv1.5 expression after silencing of RAGE were detected by Western blot analyses. **a** Representative blots showing the efficiency of RAGE silencing. Scr, scrambled siRNA (negative control); siR 1 and siR 2, two different sequences of RAGE siRNA. **b** Mean data for RAGE expression levels in CSMCs with or without RAGE silencing. **c** Representative blots showing protein expression of RAGE, Kv1.2 and Kv1.5 in CSMCs treated with Scr siRNA, 100 μg/mL AGE + Scr siRNA, RAGE siRNA or 100 μg/mL AGE + RAGE siRNA, respectively. The cells were transfected for 48 h and then incubated with or without AGE for 24 h. β-actin was used as an internal reference. **d-f** Mean data for protein expression levels. *n* = 3 per group. * *P* < 0.05 vs. Scr siRNA group; # *P* < 0.05 vs. AGE + Scr siRNA group

In cultured CSMCs, AGE significantly reduced Kv1.2/Kv1.5 expression, increased the expression of RAGE and NOX-2, and reduced PPAR-γ expression; ALA or PIO reversed these AGE-mediated effects; and GW9662 partly reversed the effects of PIO (Fig. 4). The interaction between AGE/RAGE and Kv channel impairment was further elucidated by gene transfection with RAGE siRNA in CSMCs. Western blot analyses showed that RAGE siRNA reduced the RAGE expression by more than 50%, confirming the desired efficiency of RAGE silencing (Fig. 5a-b). After being transfected for 48 h and then incubated with or without AGE for 24 h, the protein expression of Kv1.2 and Kv1.5 in CSMCs was significantly higher when co-treated with RAGE siRNA and AGE as compared with the scrambled siRNA (negative control) + AGE group (Fig. 5c-f), suggesting that silencing of RAGE alleviated the AGE-induced Kv1.2/1.5 channel impairment.

**Fig. 6:**
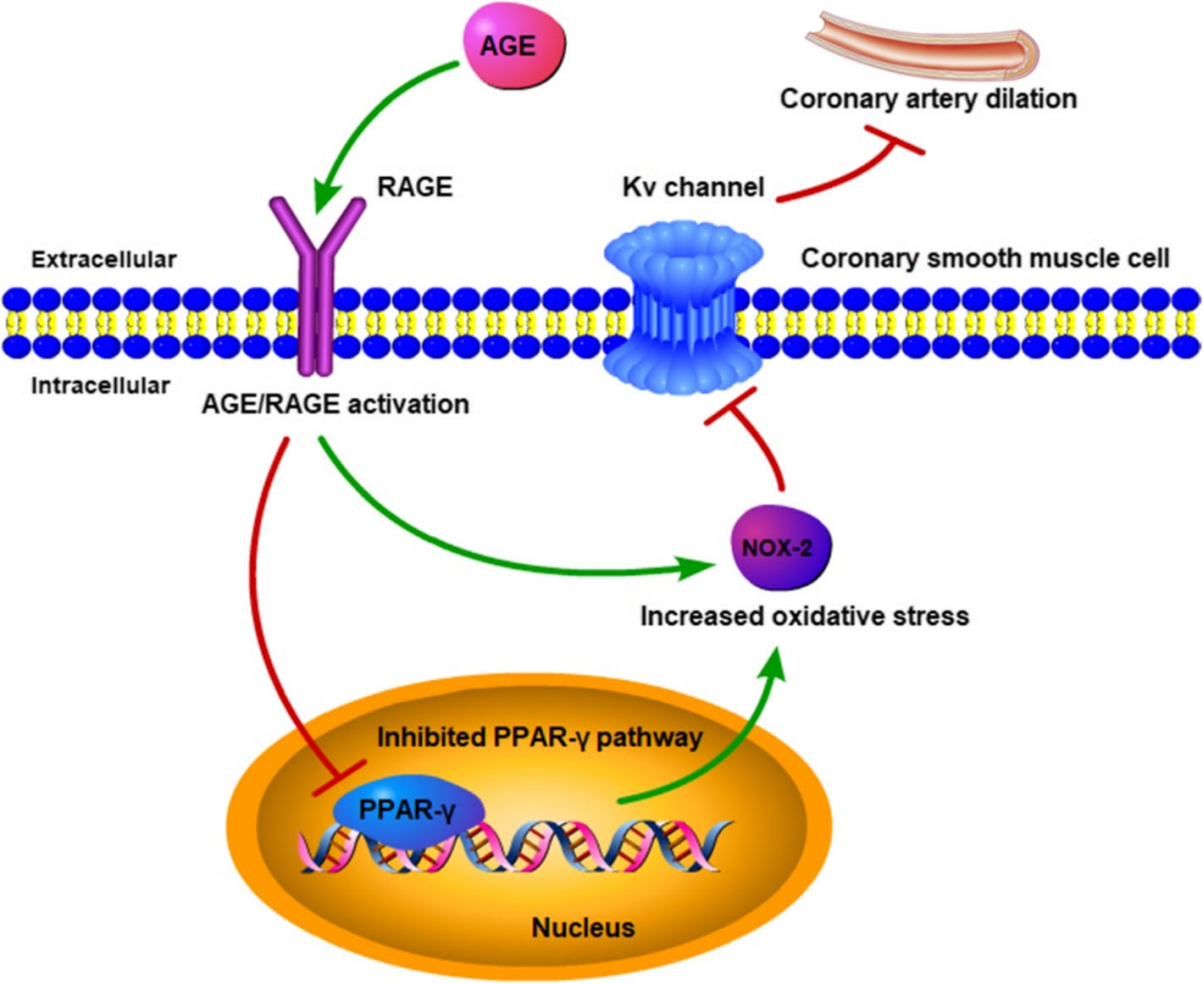
A central schematic diagram of the present study

## Discussion

Our study had two major novel findings. First, in vitro treatment with AGE reduced Kv1.2 and Kv1.5 protein expression in CSMCs and further impaired the Kv channel-mediated dilation of RSCAs in an endothelium-independent manner. Second, RAGE regulated the AGE-induced Kv channel impairment. The PPAR-γ pathway might be subsequently inhibited by AGE/RAGE activation, thereby inducing the oxidative stress-associated Kv channel damage. A central illustration of this study was shown in Fig. 6. 

In the present study, we denuded the endothelium and evaluated the Kv channel-mediated vasodilation without the influence of endothelium-derived hyperpolarizing factors. Forskolin was used as a vasodilator to induce a cAMP-mediated relaxation, which is sensitive to oxidative stress and independent of endothelium [8]. Of note, the coronary arteries were incubated for 24 h in Western blot analyses aiming to detect the alteration of target protein levels, however, the incubation time was relative short (2 h) in functional analyses because the vasomotor viability is vulnerable and should be reserved for isometric force measurement.

It has been reported that the Kv and large-conductance Ca^2+^-activated K^+^ (BK_Ca_) channels are two major types of K^+^ currents in CSMCs, and Kv channels play a predominant role in regulating coronary dilation. The expression of specific Kv subunits varies greatly among species and vascular beds, while the Shaker-related Kv1 channels are functionally significant Kv channels abundantly expressed in most animal vascular beds and are 4-AP sensitive [24]. Further, the representative Kv1.2 and Kv1.5 channels were detected because they are dominant Kv1 α-subunits and form the channel complex together in both rat and human coronary arteries. Previous studies have shown that the function, protein levels and current density of Kv1.2/1.5 channels in CSMCs could be impaired in the settings of hyperglycemia [8–10], oxidative stress [5, 11], and in diabetic animal models [11–13]. A recent article focused on the effect of AGE on K_Ca_ channel and proved that AGE damaged K_Ca_2.3 and K_Ca_3.1-mediated relaxation via oxidative stress in rat mesenteric arteries [25]. Consistent with these results, we found that treatment with AGE in vitro significantly reduced the protein expression of Kv1.2/1.5 channel in CSMCs and further impaired the Kv channel-mediated dilation in RSCAs. Meanwhile, the AGE-associated downregulation and dysfunction of Kv channel could be markedly restored by the AGE breaker (ALA), indicating the deleterious effect of AGE on the Kv channel.

RAGE is a multiligand, transmembrane receptor for AGE that expressed in numerous cell types including CSMCs. By engaging RAGE, AGE activates its downstream proinflammatory and prooxidative signaling pathways, and eventually induces a variety of vascular damages [26]. We cannot exclude the possibility that AGE could directly glycate Kv1.2/1.5 protein and thus impair its structure and function, but AGE is more likely to cross-link collagen rather than ion channels [1, 2]. Our data confirmed the interaction between AGE/RAGE axis and the Kv channel impairment. RAGE overexpression was accompanied by the impaired Kv1.2/1.5 expression in CSMCs and RSCAs exposed to AGE, while ALA largely reversed these effects. We further transfected the CSMCs with RAGE siRNA and found that the silencing of RAGE almost completely preserved the Kv1.2/1.5 protein levels. Therefore, we may conclude that the AGE-associated Kv impairment in CSMCs is primarily mediated through the activation of RAGE.

Numerous studies have proved that the activities and function of Kv channel are regulated by oxidative stress in most vascular beds [27, 28]. Meanwhile, the AGE/RAGE axis can stimulate the ROS generation and enhance the expression of NADPH oxidase subunits including NOX2, p22phox, and p40phox, thereby inducing vascular dysfunction in diabetic animal models [19, 29]. Our study also showed that AGE increased NOX-2 protein levels in CSMCs and ALA reversed these effects. The increased NOX-2 expression was accompanied by the RAGE overexpression and impaired Kv1.2/1.5 expression, suggesting that the oxidative stress might be responsible for the AGE-induced Kv impairment.

The nuclear receptor PPAR-γ is a well-known transcriptional factor that regulates glucose metabolism and adipogenesis. Activators of PPAR-γ are reported to exert anti-inflammatory, anti-oxidative, anti-fibrotic and anti-proliferative effects on vascular cells [17]. Several studies have provided the evidence that PPAR-γ signaling could be inhibited by AGE/RAGE activation [17, 18, 30]. Meanwhile, the PPAR-γ agonists conversely downregulate RAGE expression, inhibit ROS production, and finally attenuate the harmful effects of AGE in both SMC cell culture and diabetic animal models [31, 32]. Our study also showed that AGE reduced the PPAR-γ expression, and in turn, PIO decreased the RAGE expression and GW9662 reversed the inhibiting effects of PIO on RAGE expression. These data indicate a close interaction between AGE/RAGE axis and PPAR-γ pathway in CSMCs. However, ALA restored the AGE-induced downregulation of PPAR-γ in CSMCs, but this effect was not significant in incubated arteries. Actually, ALA had no direct effect on PPAR-γ, and cells tended to be sensitive to in vitro interventions than tissues. Thus, the effect of ALA on PPAR-γ might be apparent in cells, whereas in arterial tissues, the effect of 1 mM ALA might be subtle and no significantly PPAR-γ restoration was observed. In addition, PIO reduced the NOX-2 expression, restored the Kv1.2/1.5 expression and reserved the functional loss of Kv channel. Therefore, the AGE/RAGE axis may inhibit PPAR-γ pathway, and further increase oxidative stress and damage Kv channels in coronary arteries.

Several limitations of this study should be acknowledged. First, we did not use diabetic animal models, so our findings remain to be verified in in vivo experiments. Second, the 4-AP-sensitive vasodilation refers to the function of the whole Kv channel family members, yet we only focused on and detected the representative Kv1.2/1.5 channel protein expression. Ideally, a selective Kv1.2 or Kv1.5 channel blocker should be used. Besides, the current density of Kv channel was not evaluated by using patch clamp. Third, a causal relationship between oxidative stress and the AGE-induced Kv impairment was not fully elucidated because we didn’t use the ROS scavenger or NADPH oxidase inhibitor as interventions. The ROS levels in CSMCs were not detected, neither. Fourth, the interplay between AGE/RAGE and PPAR-γ pathway remains to be further clarified by genetic approaches. For instance, by inducing PPAR-γ overexpression in CSMCs and then incubating with AGE, we may better reveal how RAGE is mediated by the PPAR-γ pathway and their combined effects on the Kv channel function.

## Conclusion

Our study demonstrates that AGE decreases Kv1.2/1.5 protein expression in CSMCs and further impairs the Kv channel-mediated SMC-dependent dilation rat small coronary arteries. The AGE/RAGE axis-associated inhibition of PPAR-γ pathway and enhancement of oxidative stress may contribute to AGE-mediated Kv channel dysfunction. These results may shed light on prophylactic or therapeutic target to manage vascular dysfunction in patients with diabetes.

## Supplementary information

**Additional file 1: Supplementary Table 1.** Comparison of Forskolin-induced relaxation and Kv channel-mediated dilation in RSCAs with different treatment. **Supplementary Figure 1** The isolation of RSCAs and the mounting of arterial rings. (A)The left anterior descending artery from a normal SD rat was identified. (B) RSCAs were isolated and the endothelium was denuded with air. (C) Arterial rings were mounted with two steel wires in the chamber of a multi-myograph DMT system. **Supplementary Figure 2** Primary curves showing Forskolin-elicited vasodilation in freshly isolated RSCAs Typical records showing concentration-dependent relaxations induced by Forskolin in U46619-precontracted coronary arteries from normal SD rats. **Supplementary Figure 3** Morphology of primary CSMCs. (A)The morphology and growth characteristics of the cells in light microscopy were typical of SMCs. (B) Primary CSMCs were identified by positive α-smooth muscle actin staining using immunofluorescence. Magnification: left x100, right x400. **Supplementary Figure 4** HE staining of incubated RSCAs HE staining of cross-sectioned RSCAs after incubation. A: DMEM; B: BSA; C: AGE; D: AGE+ALA; E: AGE+PIO; F: AGE+PIO+GW9662. n=3 per group. Magnification: x200. **Supplementary Figure 5** Masson staining of incubated RSCAs Masson staining of cross-sectioned RSCAs after incubation. A: DMEM; B: BSA; C: AGE; D: AGE+ALA; E: AGE+PIO; F: AGE+PIO+GW9662. n=3 per group. Magnification: x200.

**Additional file 2: Supplementary Figure 1.** Original Western blot images of target proteins in rat small coronary arteries (Fig. 2 repeat 1 in manuscript). A: RAGE, B: Kv1.2, C: Kv1.5 & PPAR-γ, D: NOX-2, E: Actin. **Supplementary Figure 2.** Original Western blot images of target proteins in rat small coronary arteries (Fig. 2 repeat 2). A: RAGE, B: Kv1.2, C: Kv1.5, D: PPAR-γ, E: NOX-2, F: Actin. **Supplementary Figure 3.** Original Western blot images of target proteins in cultured cells (Fig. 4 repeat 1 in manuscript). A: RAGE, B: Kv1.2, C: Kv1.5, D: PPAR-γ, E: NOX-2, F: Actin. **Supplementary Figure 4.** Original Western blot images of target proteins in cultured cells (Fig. 4 repeat 2). A: RAGE, B: Kv1.2, C: Kv1.5, D: PPAR-γ, E: NOX-2, F: Actin. **Supplementary Figure 5.** Original Western blot images of target proteins in cultured cells (Fig. 4 repeat 3). A: RAGE, B: Kv1.2, C: Kv1.5, D: PPAR-γ, E: NOX-2, F: Actin. **Supplementary Figure 6.** Original Western blot images of RAGE siRNA experiment in cultured cells (Fig.5 repeat 1 in manuscript). A: RAGE test, B: Actin for RAGE, C: RAGE, D: Kv1.2, E: Actin. **Supplementary Figure 7.** Original Western blot images of RAGE siRNA experiment in cultured cells (Fig. 5 repeat 2&3). A: RAGE test (repeat 2), B: Action for RAGE (repeat 2), C: RAGE (repeat 2), D: RAGE test (repeat 3), E: Action for RAGE (repeat 3), F: RAGE (repeat 3), G: Kv1.2 (repeat 23), H: Kv1.5 (repeat 2&3), I: Action (repeat 2&3).

## Data Availability

The datasets used and/or analyzed during the current study available from the corresponding author on reasonable request.

## Abbreviations

AGE: Advanced glycation end products
RAGE: Receptor for AGE
RSCAs: Rat small coronary arteries
CSMCs: Coronary artery smooth muscle cells
VSMCs: Vascular artery smooth muscle cells
PPAR-γ: Peroxisome proliferators-activated receptor-gamma
Kv: Voltage-gated K+ channel
NOX-2: Nicotinamide adenine dinucleotide phosphate (NADPH) oxidase-2
ROS: Reactive oxygen species
DMEM: Dulbecco’s modified Eagle’s medium
BSA: Bovine serum albumin
FBS: Fetal bovine serum
ALA: Alagrebrium
PIO: Pioglitazone
4-AP: 4-aminopyridine
cAMP: Cyclic adenosine monophosphate
