# Supplementary material for: Role of peroxisome proliferators-activated receptor-gamma in advanced glycation end product-mediated functional loss of voltage-gated potassium channel in rat coronary arteries
Source: BMC Cardiovasc Disord. 2020 Jul 14;20:337. doi: 10.1186/s12872-020-01613-y (PMC7362521; doi:10.1186/s12872-020-01613-y)
Supplement: Supplementary file 1 — Additional file 1: Supplementary Table 1. Comparison of Forskolin-induced relaxation and Kv channel-mediated dilation in RSCAs with different treatment. Supplementary Figure 1 The isolation of RSCAs and the mounting of arterial rings. (A)The left anterior descending artery from a normal SD rat was identified. (B) RSCAs were isolated and the endothelium was denuded with air. (C) Arterial rings were mounted with two steel wires in the chamber of a multi-myograph DMT system. Supplementary Figure 2 Primary curves showing Forskolin-elicited vasodilation in freshly isolated RSCAs Typical records showing concentration-dependent relaxations induced by Forskolin in U46619-precontracted coronary arteries from normal SD rats. Supplementary Figure 3 Morphology of primary CSMCs. (A)The morphology and growth characteristics of the cells in light microscopy were typical of SMCs. (B) Primary CSMCs were identified by positive α-smooth muscle actin staining using immunofluorescence. Magnification: left x100, right x400. Supplementary Figure 4 HE staining of incubated RSCAs HE staining of cross-sectioned RSCAs after incubation. A: DMEM; B: BSA; C: AGE; D: AGE+ALA; E: AGE+PIO; F: AGE+PIO+GW9662. n=3 per group. Magnification: x200. Supplementary Figure 5 Masson staining of incubated RSCAs Masson staining of cross-sectioned RSCAs after incubation. A: DMEM; B: BSA; C: AGE; D: AGE+ALA; E: AGE+PIO; F: AGE+PIO+GW9662. n=3 per group. Magnification: x200. [file 12872_2020_1613_MOESM1_ESM.docx]

**Supplementary Materials of Manuscript “Role of Peroxisome Proliferators-Activated Receptor-gamma in Advanced Glycation End Product-mediated Functional Loss of Voltage-gated Potassium Channel in Rat Coronary Arteries”**

**Supplementary Table 1 Comparison of Forskolin-induced relaxation and Kv channel-mediated dilation in RSCAs with different treatment.**

| Max dilation (%) | Control | BSA | AGE | AGE+ALA | AGE+PIO | AGE+PIO  +GW9662 |
| --- | --- | --- | --- | --- | --- | --- |
| Forskolin-induced dilation | | | | | | |
| 1.0*10^-7^ M | 39.5± 8.1% | 34.8±8.0% | 13.1±5.3% * | 23.1±7.3% ^#^ | 30.3±7.0% ^#^ | 20.3±6.0% ^§^ |
| 1.0*10^-6^ M | 79.1± 10.4% | 76.6±10.5% | 36.1±12.5% * | 63.0±8.2% ^#^ | 69.1±9.3% ^#^ | 47.0±9.0% ^§^ |
| Kv channel-mediated dilation | | | | | | |
| 1.0*10^-7^ M | 24.5± 6.3% | 23.3±6.6% | 9.1±3.3% * | 18.0±6.7% ^#^ | 20.6±7.4% ^#^ | 13.8±4.9 ^§^ |
| 1.0*10^-6^ M | 49.6± 8.2% | 45.3±8.7% | 18.8±8.8% * | 39.0±9.5% ^#^ | 43.5±9.0% ^#^ | 25.6±7.0 ^§^ |

The maximum dilation (%) values of general dilation and Kv channel-mediated dilation in response to 10^-7^ M and 10^-6^ M Forskolin were compared in endothelium-denuded rat small coronary arteries incubated with control vehicle (HEPES), 200μg/mL BSA, 200μg/mL AGE, 200μg/mL AGE+1mM ALA, 200μg/mL AGE+0.1mM PIO, or 200μg/mL AGE+0.1mM PIO +0.1mM GW9662 for 2h. The differences of dilation to 10^-10^-10^-8^ M Forskolin between groups were not significant and data were not shown. Other data were expressed as mean± standard deviation. n=6 vessels per group. * P<0.05 vs. BSA; # P<0.05 vs. AGE; § P<0.05 vs. AGE+PIO.

**
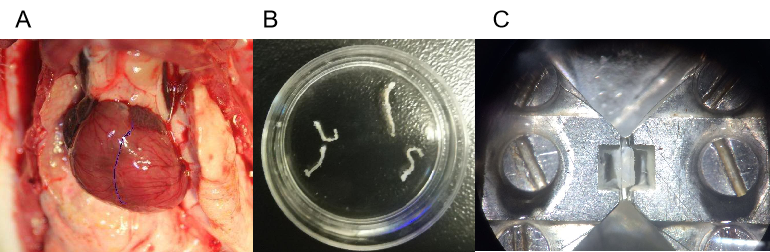
**

**Supplementary Figure 1 The isolation of RSCAs and the mounting of arterial rings**

(A)The left anterior descending artery from a normal SD rat was identified. (B) RSCAs were isolated and the endothelium was denuded with air. (C) Arterial rings were mounted with two steel wires in the chamber of a multi-myograph DMT system.

**
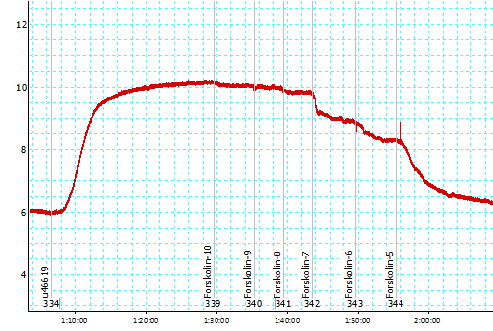
**

**Supplementary Figure 2 Primary curves showing Forskolin-elicited vasodilation in freshly isolated RSCAs**

Typical records showing concentration-dependent relaxations induced by Forskolin in U46619-precontracted coronary arteries from normal SD rats.


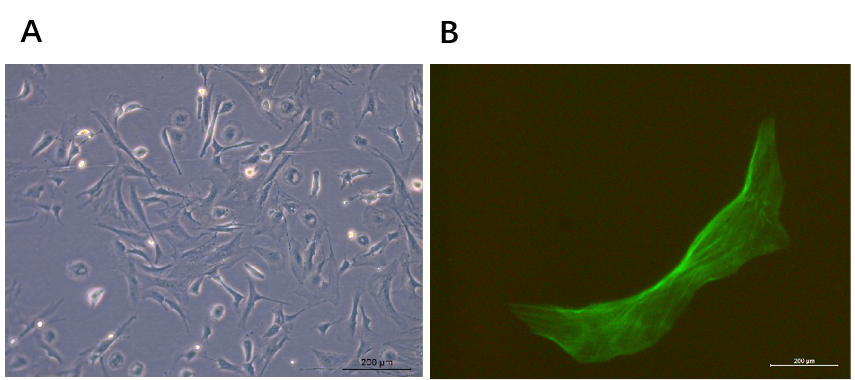


**Supplementary Figure 3 Morphology of primary CSMCs**

(A)The morphology and growth characteristics of the cells in light microscopy were typical of SMCs. (B) Primary CSMCs were identified by positive α-smooth muscle actin staining using immunofluorescence. Magnification: left x100, right x400.


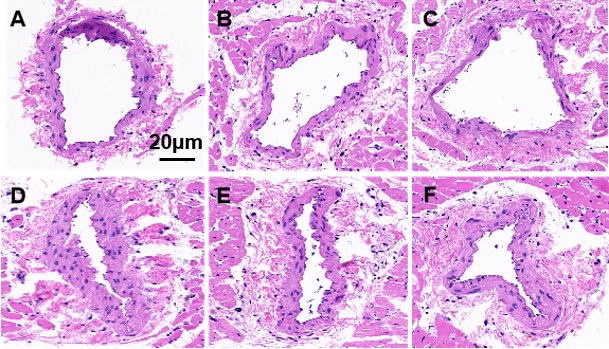


**Supplementary Figure 4 HE staining of incubated RSCAs**

HE staining of cross-sectioned RSCAs after incubation. A: DMEM; B: BSA; C: AGE; D: AGE+ALA; E: AGE+PIO; F: AGE+PIO+GW9662. n=3 per group. Magnification: x200.


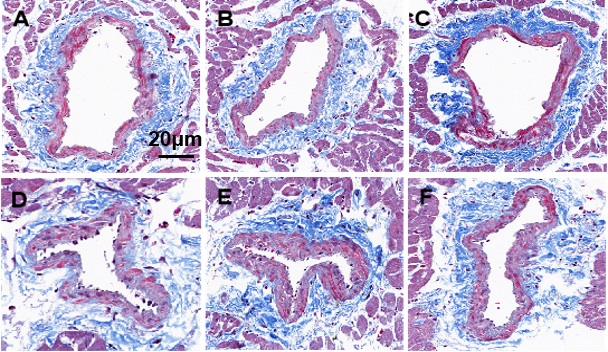


**Supplementary Figure 5 Masson staining of incubated RSCAs**

Masson staining of cross-sectioned RSCAs after incubation. A: DMEM; B: BSA; C: AGE; D: AGE+ALA; E: AGE+PIO; F: AGE+PIO+GW9662. n=3 per group. Magnification: x200.
