# Supplementary material for: Role of peroxisome proliferators-activated receptor-gamma in advanced glycation end product-mediated functional loss of voltage-gated potassium channel in rat coronary arteries
Source: BMC Cardiovasc Disord. 2020 Jul 14;20:337. doi: 10.1186/s12872-020-01613-y (PMC7362521; doi:10.1186/s12872-020-01613-y)
Supplement: Supplementary file 2 — Additional file 2: Supplementary Figure 1. Original Western blot images of target proteins in rat small coronary arteries (Fig. 2 repeat 1 in manuscript). A: RAGE, B: Kv1.2, C: Kv1.5 & PPAR-γ, D: NOX-2, E: Actin. Supplementary Figure 2. Original Western blot images of target proteins in rat small coronary arteries (Fig. 2 repeat 2). A: RAGE, B: Kv1.2, C: Kv1.5, D: PPAR-γ, E: NOX-2, F: Actin. Supplementary Figure 3. Original Western blot images of target proteins in cultured cells (Fig. 4 repeat 1 in manuscript). A: RAGE, B: Kv1.2, C: Kv1.5, D: PPAR-γ, E: NOX-2, F: Actin. Supplementary Figure 4. Original Western blot images of target proteins in cultured cells (Fig. 4 repeat 2). A: RAGE, B: Kv1.2, C: Kv1.5, D: PPAR-γ, E: NOX-2, F: Actin. Supplementary Figure 5. Original Western blot images of target proteins in cultured cells (Fig. 4 repeat 3). A: RAGE, B: Kv1.2, C: Kv1.5, D: PPAR-γ, E: NOX-2, F: Actin. Supplementary Figure 6. Original Western blot images of RAGE siRNA experiment in cultured cells (Fig.5 repeat 1 in manuscript). A: RAGE test, B: Actin for RAGE, C: RAGE, D: Kv1.2, E: Actin. Supplementary Figure 7. Original Western blot images of RAGE siRNA experiment in cultured cells (Fig. 5 repeat 2&3). A: RAGE test (repeat 2), B: Action for RAGE (repeat 2), C: RAGE (repeat 2), D: RAGE test (repeat 3), E: Action for RAGE (repeat 3), F: RAGE (repeat 3), G: Kv1.2 (repeat 23), H: Kv1.5 (repeat 2&3), I: Action (repeat 2&3). [file 12872_2020_1613_MOESM2_ESM.docx]

**Supplementary Data for Original Western Blot Images of the Manuscript**


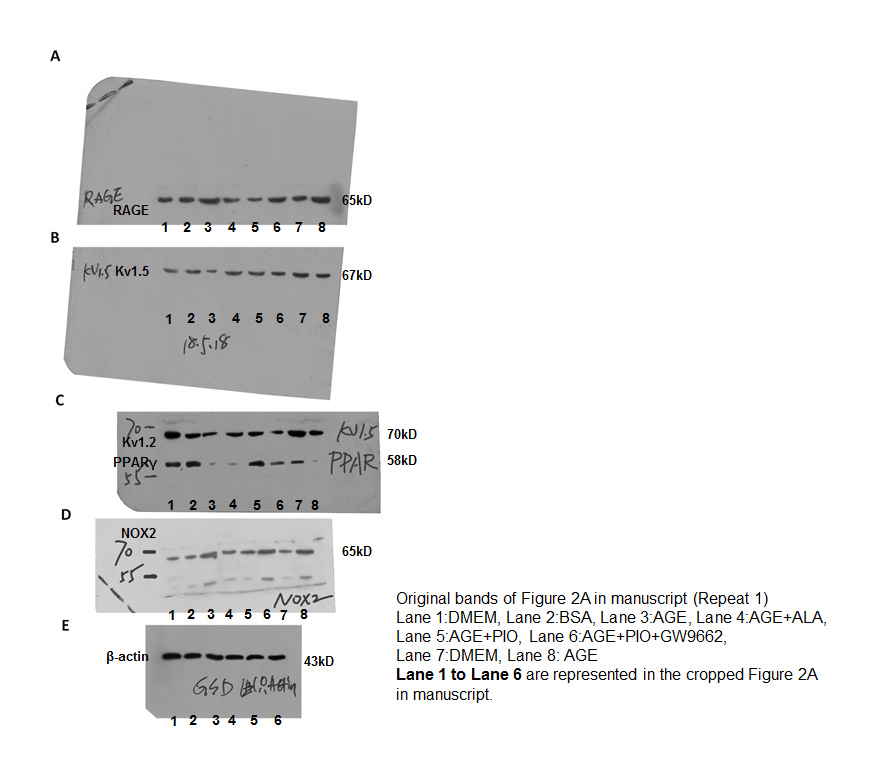


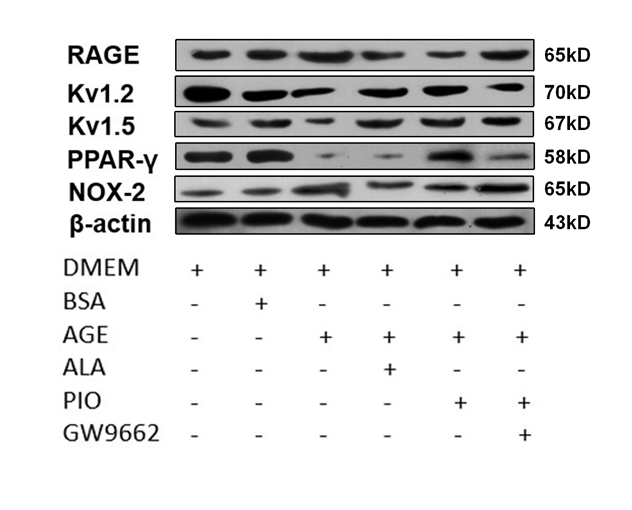


Supplementary Figure 1. Original Western blot images of target proteins in rat small coronary arteries (Fig.2 repeat 1 in manuscript). A: RAGE, B: Kv1.2, C: Kv1.5 & PPAR-γ, D: NOX-2, E: Actin.


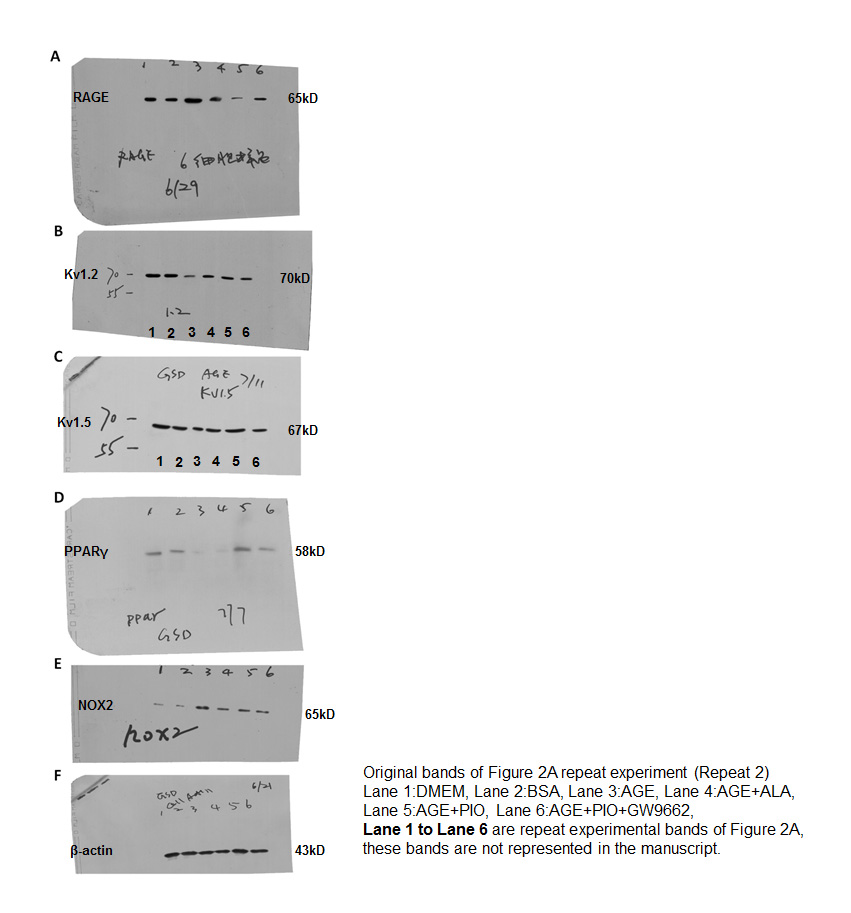


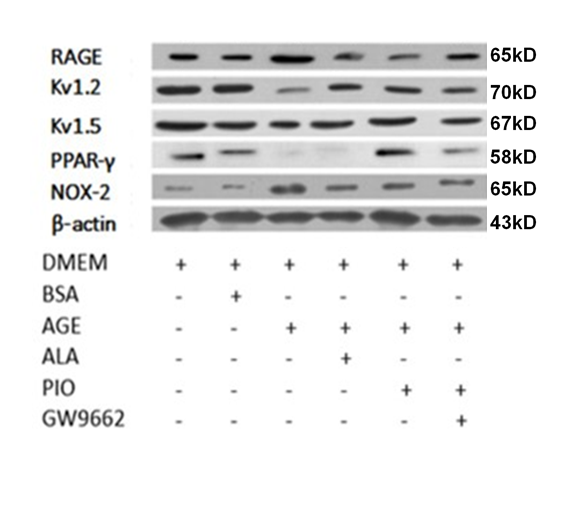


Supplementary Figure 2. Original Western blot images of target proteins in rat small coronary arteries (Fig.2 repeat 2). A: RAGE, B: Kv1.2, C: Kv1.5, D: PPAR-γ, E: NOX-2, F: Actin.


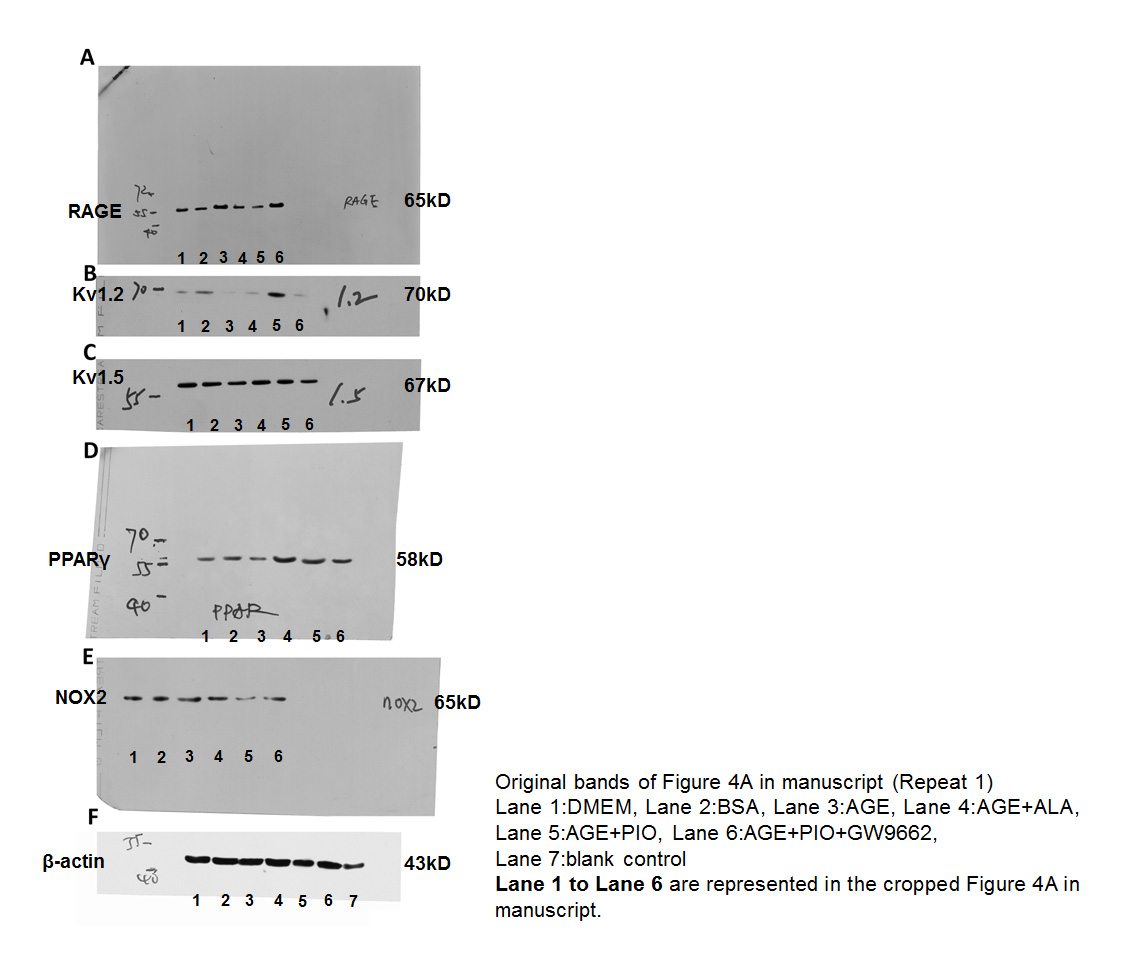


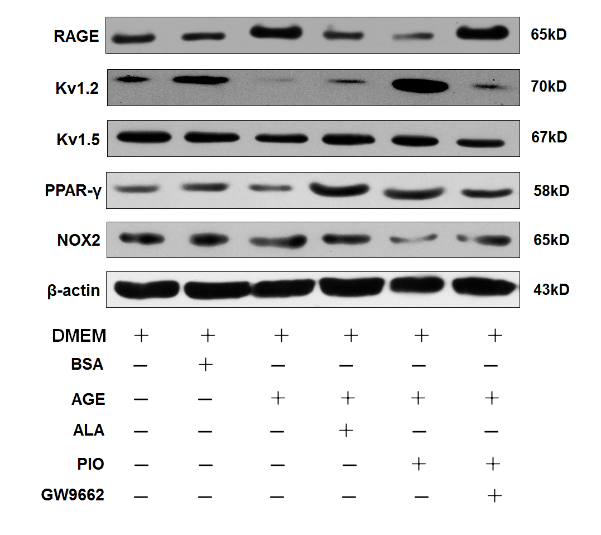


Supplementary Figure 3. Original Western blot images of target proteins in cultured cells (Fig.4 repeat 1 in manuscript). A: RAGE, B: Kv1.2, C: Kv1.5, D: PPAR-γ, E: NOX-2, F: Actin.


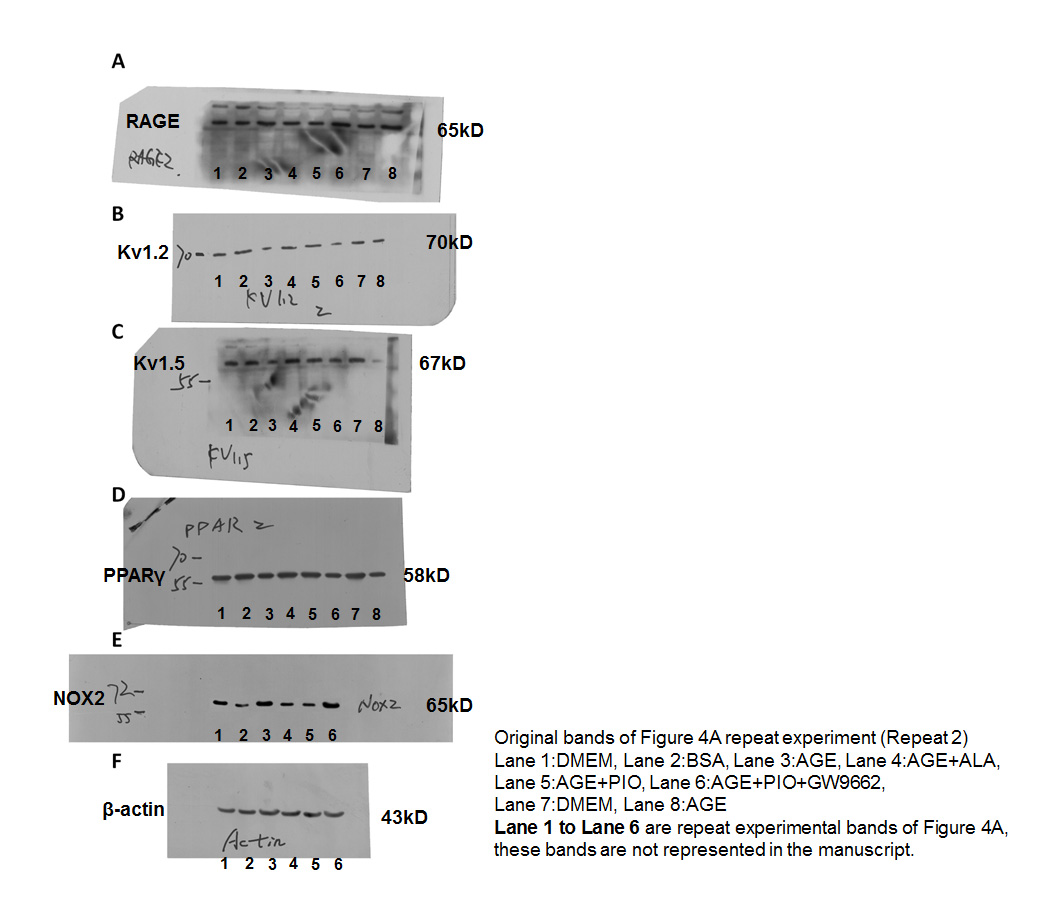


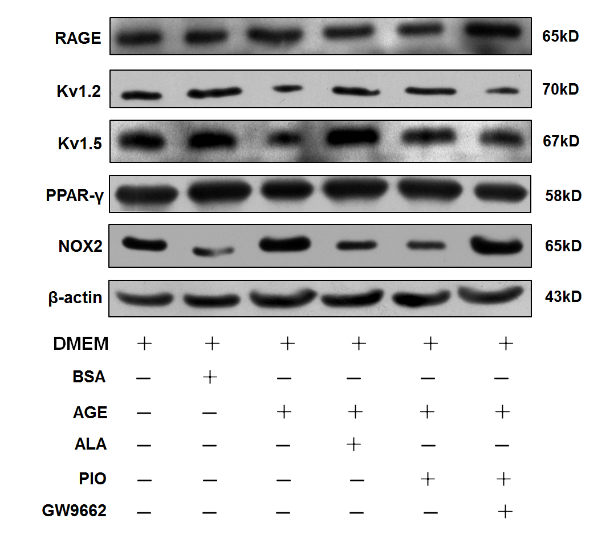


Supplementary Figure 4. Original Western blot images of target proteins in cultured cells (Fig.4 repeat 2). A: RAGE, B: Kv1.2, C: Kv1.5, D: PPAR-γ, E: NOX-2, F: Actin.


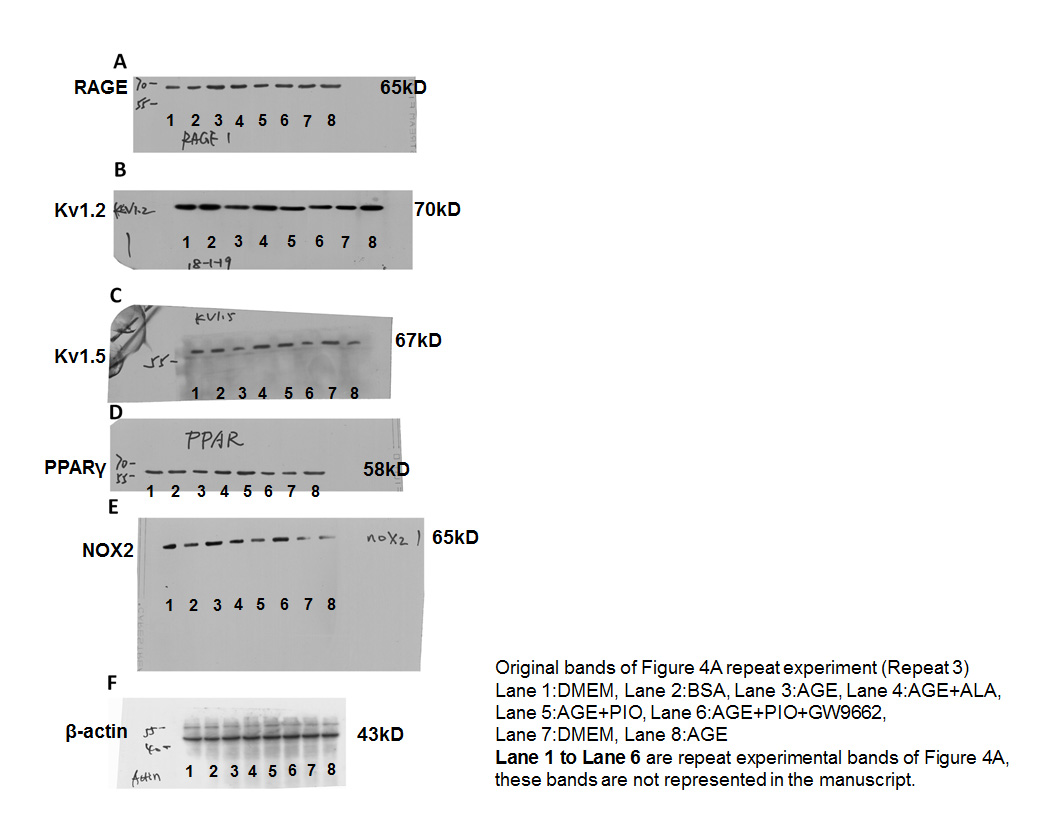


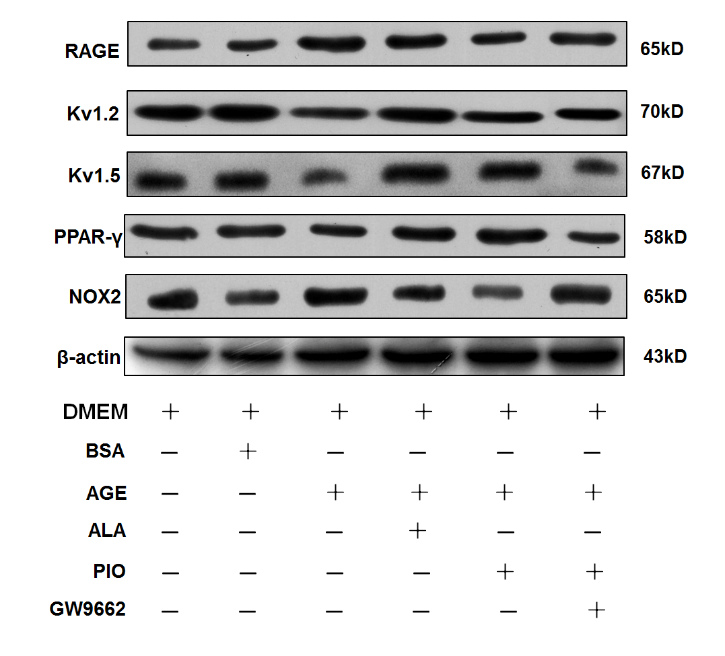


Supplementary Figure 5. Original Western blot images of target proteins in cultured cells (Fig.4 repeat 3). A: RAGE, B: Kv1.2, C: Kv1.5, D: PPAR-γ, E: NOX-2, F: Actin.


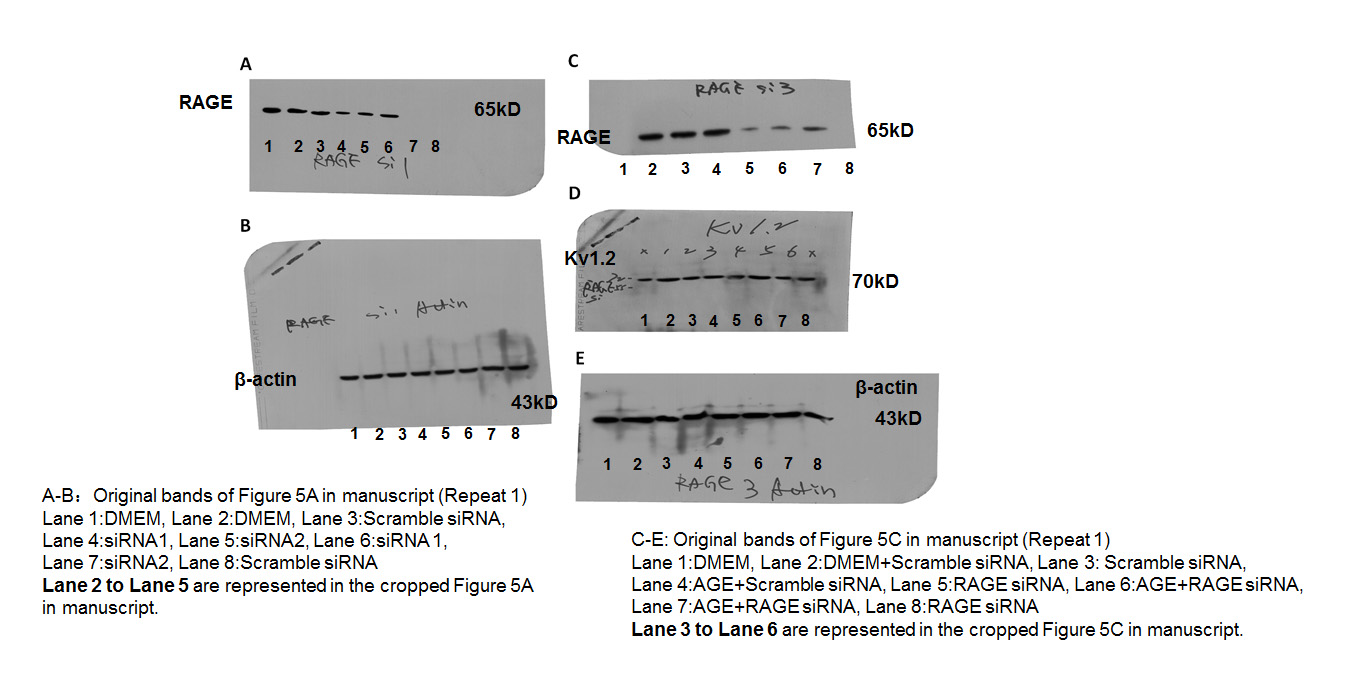


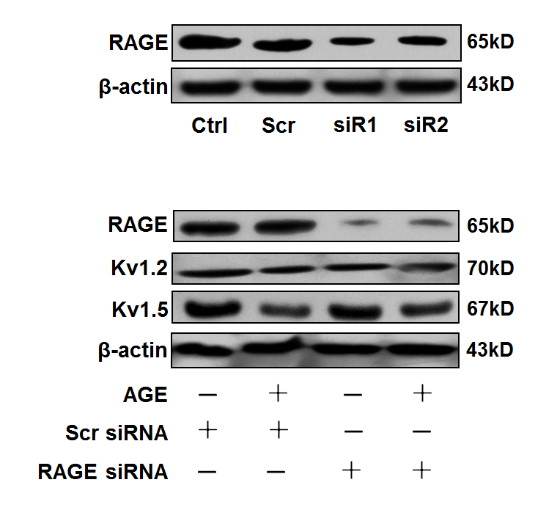


Supplementary Figure 6. Original Western blot images of RAGE siRNA experiment in cultured cells (Fig.5 repeat 1 in manuscript). A: RAGE test, B: Actin for RAGE, C: RAGE, D: Kv1.2, E: Actin.


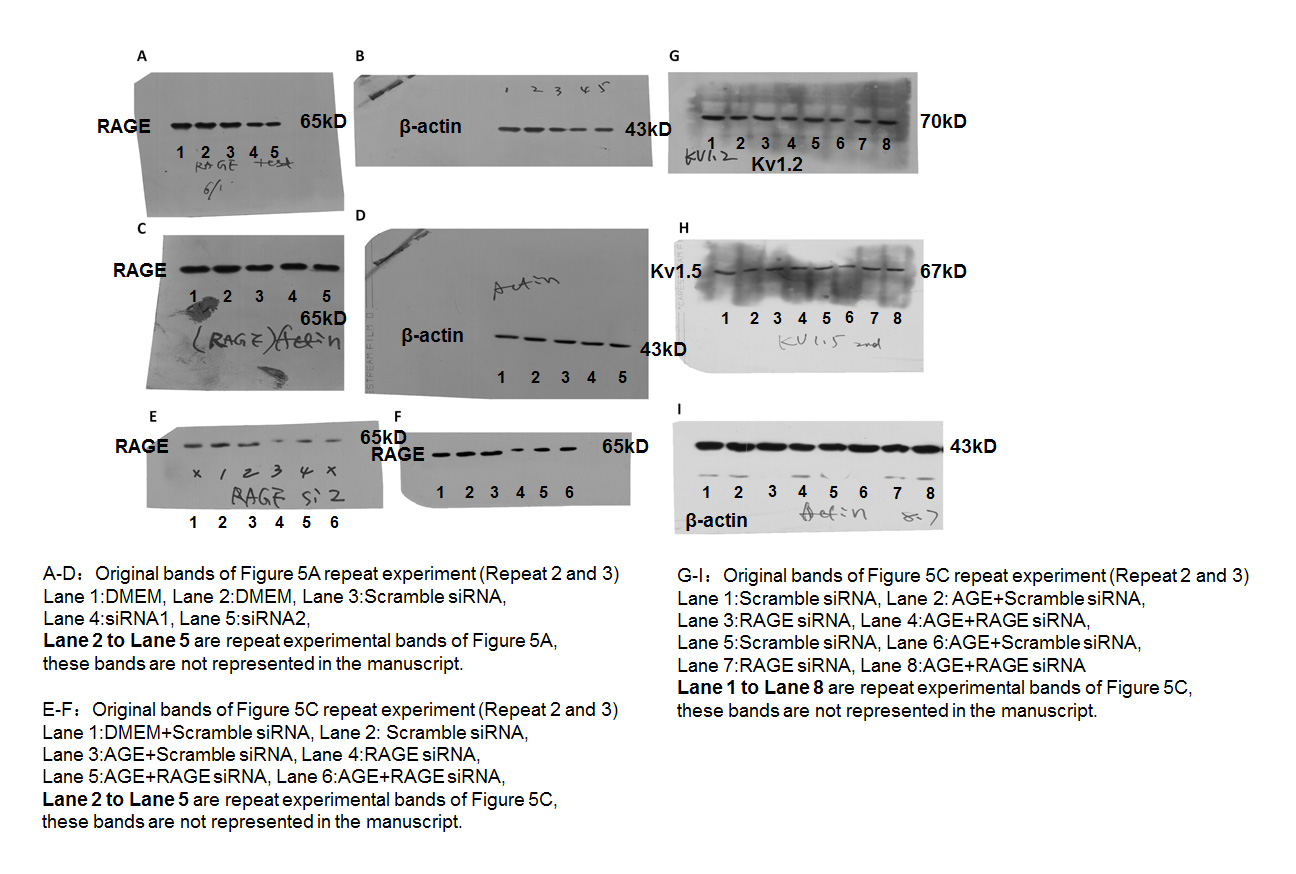


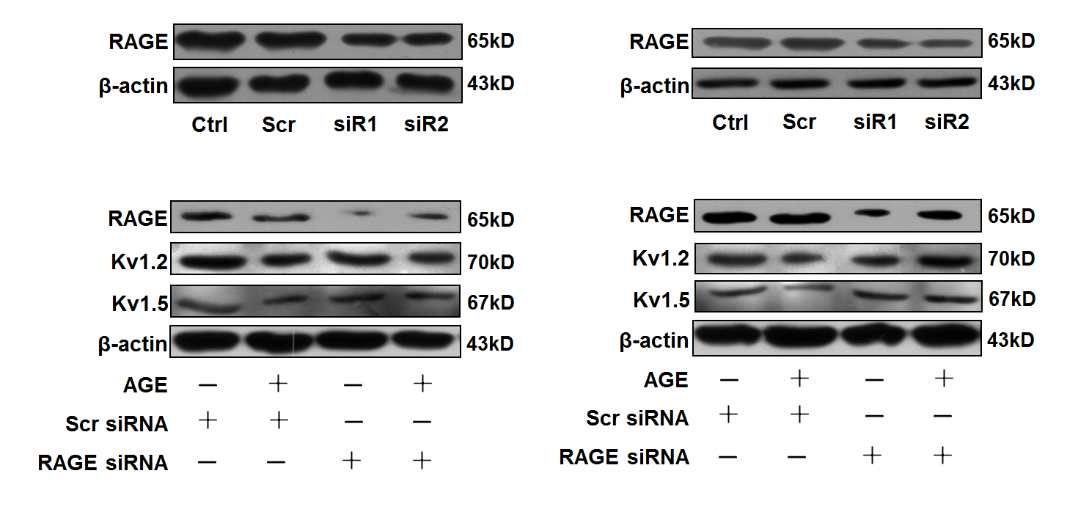


Supplementary Figure 7. Original Western blot images of RAGE siRNA experiment in cultured cells (Fig.5 repeat 2&3). A: RAGE test (repeat 2), B: Action for RAGE (repeat 2), C: RAGE (repeat 2), D: RAGE test (repeat 3), E: Action for RAGE (repeat 3), F: RAGE (repeat 3), G: Kv1.2 (repeat 2&3), H: Kv1.5 (repeat 2&3), I: Action (repeat 2&3).
